# Ectopic blastema induction by nerve deviation and skin wounding: a new regeneration model in *Xenopus laevis*

**DOI:** 10.1002/reg2.11

**Published:** 2014-05-28

**Authors:** Kazumasa Mitogawa, Ayako Hirata, Miyuki Moriyasu, Aki Makanae, Shinichirou Miura, Tetsuya Endo, Akira Satoh

**Affiliations:** ^1^ Okayama University, Research Core for Interdisciplinary Sciences3‐1‐1 TsushimanakaKitakuOkayama700‐8530Japan; ^2^ Division of Liberal Arts, Aichi Gakuin UniversityNissinAichi470‐0195Japan

**Keywords:** Accessory limb model (ALM), blastema, limb regeneration, nerve, *Xenopus laevis*

## Abstract

Recently, the accessory limb model (ALM) has become an alternative study system for limb regeneration studies in axolotls instead of using an amputated limb. ALM progresses limb regeneration study in axolotls because of its advantages. To apply and/or to compare knowledge in axolotl ALM studies to other vertebrates is a conceivable next step. First, *Xenopus laevis*, an anuran amphibian, was investigated. A *Xenopus* frog has hypomorphic regeneration ability. Its regeneration ability has been considered intermediate between that of non‐regenerative higher vertebrates and regenerative urodele amphibians. Here, we successfully induced an accessory blastema in *Xenopus* by skin wounding and rerouting of brachial nerve bundles to the wound site, which is the regular ALM surgery. The induced *Xenopus* ALM blastemas have limited regenerative potential compared with axolotl ALM blastemas. Comparison of ALM blastemas from species with different regenerative potentials may facilitate the identification of the novel expression programs necessary for the formation of cartilage and other tissues during limb regeneration.

## Introduction

Urodele amphibians show much greater regenerative capacity than higher vertebrates. For example, axolotl, a urodele amphibian, can regenerate limbs after amputation. This phenomenon has long been studied as a model to elucidate regenerative mechanisms, which are present in amphibians but not in higher vertebrates. Anuran amphibians may be another valuable model organism because their regenerative phenotype is intermediate between that of urodele amphibians and higher vertebrates. Mature *Xenopus laevis*, for example, exhibits an initial limb regeneration response but cannot restore the entire structure (Sessions and Bryant 1988; Suzuki et al. 2006). Instead, the regenerated tissue is a hypomorphic cone‐shaped cartilaginous structure called a spike. The spike has two major defects in limb organization, a pattern defect and the absence of tissues such as muscles (Endo et al. 2000; Satoh et al. 2005). It exhibits neither joint formation nor branching but possesses certain limb features such as nails (Satoh et al. 2006) and nuptial pads (Tassava 2004) used for mating in male frogs. Thus, *Xenopus* retains partial regenerative capacity and thus is a valuable model to study evolutionary changes in regenerative mechanisms.

*Xenopus* blastema formation depends on the presence of residual nervous tissue (Endo et al. 2000; Cannata et al. 2001); denervation of limb stump results in failure of blastema induction, a response shared by urodele amphibians. Thus, the initial progression of *Xenopus* limb regeneration appears to be similar to that of urodele amphibians. The amputated surface is soon covered with migrating epithelial cells that form a wound epithelium, which later differentiates into a regeneration‐specific epithelium called an apical epithelial/epithelium cap (AEC). Blastema cells accumulate under the AEC and express embryonic marker genes such as *Msx1*, *Fgf10*, *Fgf8*, *Prrx1* (also referred to as *Prx1*), *Hoxa13*, *Hoxa11*, and *Tbx5* (Koshiba et al. 1998; Endo et al. 2000; Yokoyama et al. 2000; Suzuki et al. 2005; Ohgo et al. 2010), and the presence of residual nerve tissue is thought to maintain the expression of these genes, which maintain blastema cells in an undifferentiated state. In the absence of nerves, the amputated limb does not grow a blastema and wound healing takes place instead. Thus, as in urodele amphibians, the presence of residual nerve tissue is the key to successful limb regeneration in *Xenopus*.

To investigate the early mechanisms of limb regeneration, a new model, the accessory limb model (ALM), was developed in axolotls (Endo et al. 2000) and was demonstrated to have several advantages over limb amputation (Makanae and Satoh 2012). One of the most prominent advantages of the ALM is that only nerves contacting a skin wound are necessary for blastema development. In other words, it is possible to study early regenerative processes by focusing on the interactions between these two tissues alone. In the ALM, a small skin wound is created without damaging deeper tissues, such as muscles. Next, nerves are rerouted to the wound. The surrounding epidermis, called the wound epidermis or wound epithelium, migrates to heal the open wound (Satoh et al. 2008). The rerouted nerves are located just under the wound epithelium and can interact with it to induce AEC formation. AEC and nerves create a regenerative environment to induce blastema cell differentiation in order to form various limb tissues (Makanae and Satoh 2012). Many of these blastema cells are derived from skin dermis (Muneoka and Bryant 1984a,b; Gardiner et al. 1986; Endo et al. 2004; Kragl et al. 2009; Hirata et al. 2010). Such skin‐derived blastema cells accumulate around the point of the AEC–nerve interaction, leading to the formation of a domed blastema. The simplicity of this model allows for detailed molecular analysis.

Is the ALM possible only in regenerative animals? In the case of axolotls, skin wounding plus nerve rerouting to the wound site are sufficient to induce limb regeneration response (Endo et al. 2004). Moreover, molecules have been identified that can substitute for nerve tissues (Kumar et al. 2007; Satoh et al. 2011; Makanae et al. 2013). To apply these findings to the problem of regeneration in higher vertebrates, it is first necessary to study accessory limb development in transition species such as *Xenopus*. Nerve and skin are easily identifiable tissues, and the molecular mechanisms of their development have been extensively studied. It may be possible to create similar responses in other species through the interaction of skin and nerve. As a first step, in this study we applied the ALM methodology to *Xenopus laevis*.

## Results

### Excessive nerve rerouting into a skin wound gives rise to an accessory blastema in *Xenopus*

**Figure 1:**
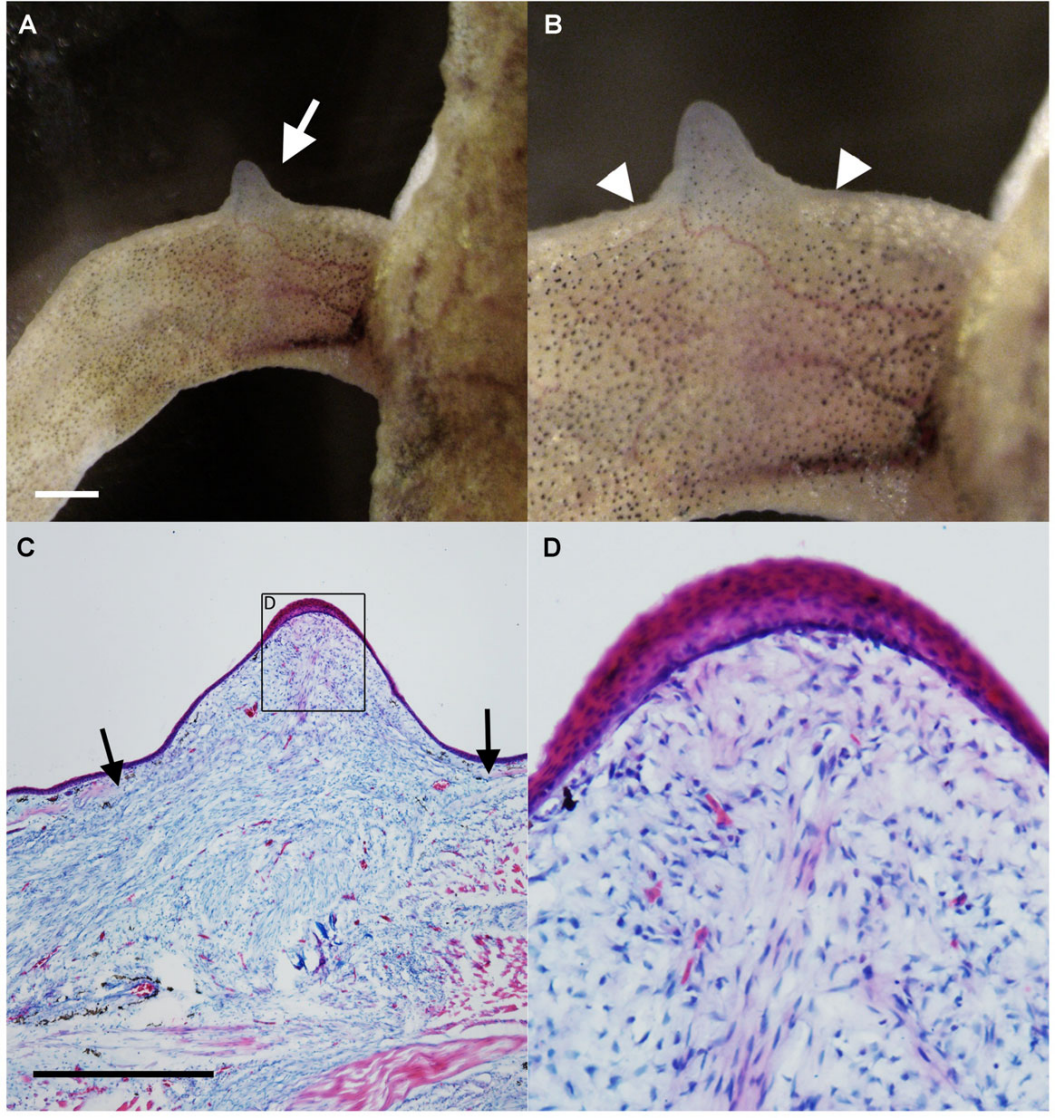
ALM blastema‐like formation (bump) in *Xenopus laevis*. (A, B) Nerve deviation to the skin wound resulted in induction of the bump. Arrow in A indicates the induced bump. Arrowheads in B indicate the border of the wounded skin. (C, D) Histological observations. (C) In the bump region, many mesenchymal cells can be observed. Arrows indicate the border of the dermal collagen layer. (D) Higher magnification of (C). The distal epithelium, which is expected to be an AEC, was thickened. Scale bars in (A) and (C) are 1 mm.

We first examined whether the same procedure used to induce ectopic blastemas in axolotls could be successfully applied to *Xenopus*. A piece of skin was removed using forceps and scissors, and the nerves were rerouted to the wound on the same day. After the rerouted nerve was in place, the animal was placed on ice for 3 h and then maintained in dechlorinated water during observation. In axolotls, a single nerve bundle is often sufficient to induce ectopic blastema formation. Therefore, a single nerve bundle was rerouted to the wound in initial *Xenopus* experiments. However, this procedure did not give rise to a blastema in any of the cases (Table 1). Previous studies suggest that the amount of nerve tissue is important for *Xenopus* limb regeneration (Yokoyama et al. 2011), so subsequent attempts rerouted three major brachial nerve bundles from the *Xenopus* upper forearm to the wound site. A small blastema‐like formation (bump) with obvious melanophores was observed 2 weeks after the surgery (Table 1, Fig. 1A, B). To investigate the microstructure and histology of the *Xenopus* bump, sections were prepared and stained with hematoxylin and eosin (Fig. 1C, D). Many mononuclear cells were observable in the induced structure, which is a typical structure of a blastema. There were no glands in the bump, suggesting that the induced structure was undifferentiated tissue. The overlying epithelium was thickened, which is a typical feature of an AEC (Fig. 1D).

**Table 1:** Induction rate of ectopic spike and bump formation

Experiments	Bump formation	Spike formation	No response	Total number
ALM^1^ (1 major nerve)	0	0	25	25
ALM^1^ (3 major nerves)	16^2^	0	17	33
ALM^1^ + humerus damage	14	10	3	17
ALM − skin grafting	8	0	8	16

^1^Nerve deviation + skin wounding + skin graft from the contralateral side of the limb.

^2^Some were extended but absence of cartilaginous cells was confirmed by Alcian blue staining.

**Figure 2:**
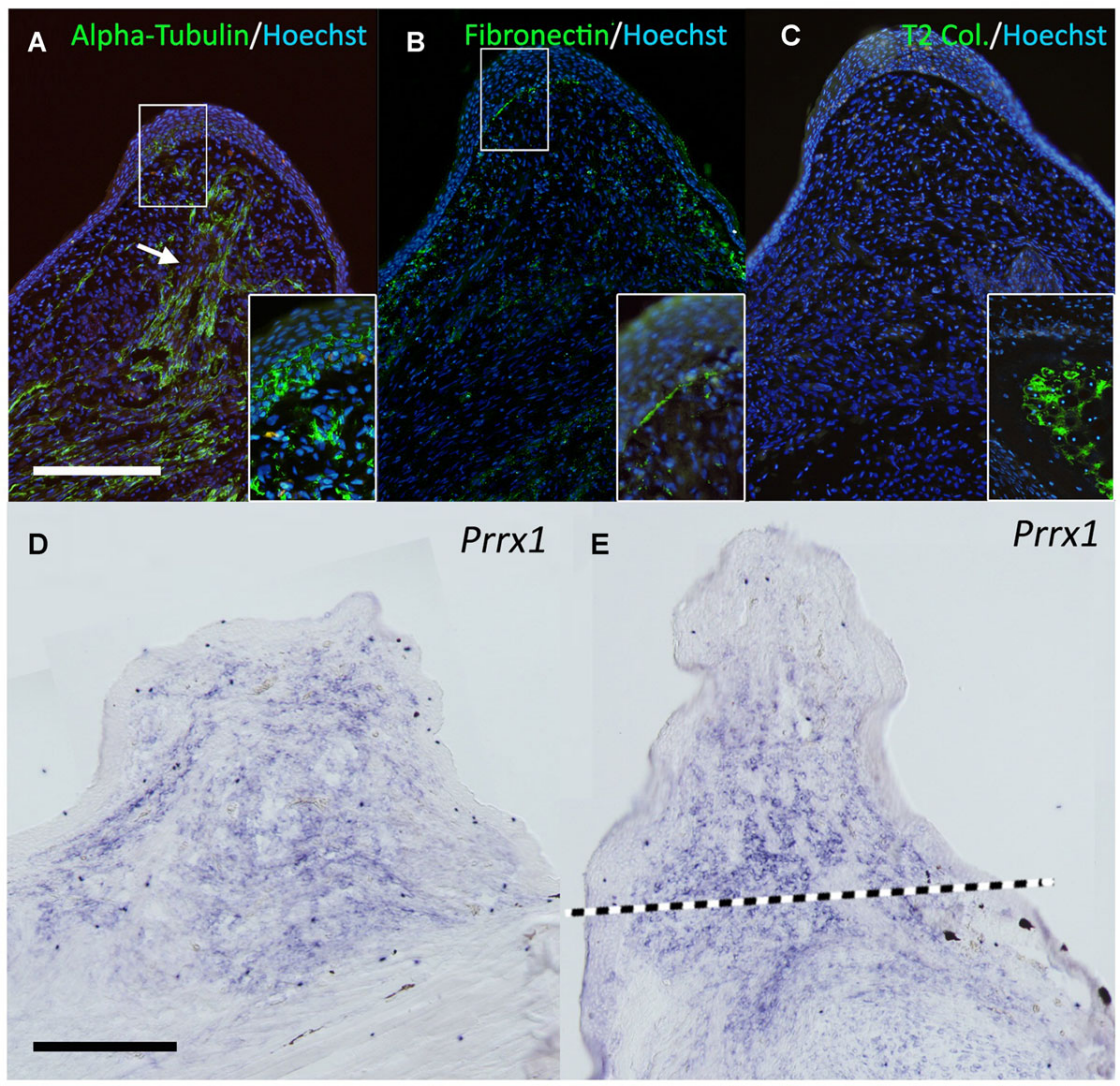
Gene expression pattern in the bump. (A) Neural tubulin (acetylated alpha‐tubulin) was visualized by immunofluorescence. The white arrow indicates the thick nerve bundle observed in the center of the bump. The deviated nerves abundantly penetrate into the overlying epithelium (inset). (B) One of the classical blastema marker genes, fibronectin, was investigated. The signal of fibronectin can be seen in the blastemal region and the epithelium−mesenchyme boundary shows intense fibronectin signal (inset). (C) Type II collagen expression. Type II collagen is a cartilage marker gene and negative in the bump. The inset shows the signal in the limb cartilage located in the proximal region as the positive control. (D, E) *Prrx1* expression. *Prrx1* can be seen in the blastemal mesenchyme in the ALM blastema (D) and the regular blastema in the amputated limb (E). The dotted line in (E) indicates the presumptive amputation plane. All conditions of the in situ hybridization were the same in (D) and (E). Scale bars in (A) and (D) are 0.5 mm.

To investigate the induced bump further, sections were analyzed. To visualize the rerouted nerves, fluorescence immunostaining was performed (Fig. 2A). A thick nerve bundle was observed near the center of the bump (Fig. 2A, arrow), and axonal penetration was observed at the distal region of the AEC (Fig. 2A, inset). Fibronectin, a typical blastema marker observed after limb amputation (Christensen and Tassava 2000), was also detected in these *Xenopus* bumps (Fig. 2B), particularly at the border (Fig. 2B, inset). Type II collagen is expressed in cartilaginous cells and *Xenopus* blastemas formed after limb amputation develop cartilaginous spikes. However, the bump induced by the ALM procedure was type II collagen‐negative (Fig. 2C). In *Xenopus*, *Prrx1* expression is a marker of blastema cells (Suzuki et al. 2005) and was expressed in the mesenchymal cells of the bump (Fig. 2D) as in the regular blastema on the amputated limb (Fig. 2E). These results suggest that the induced bump is a blastema.

**Figure 3:**
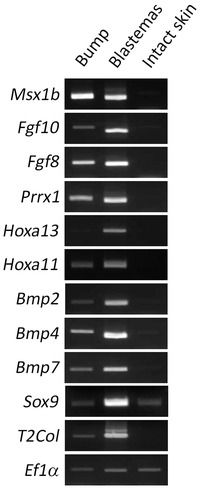
RT‐PCR analysis of the induced bump. Gene expression pattern was investigated by RT‐PCR. Samples were prepared from the induced bumps (2 weeks after the surgery), regular blastemas (MB; 2 weeks after amputation), and intact skin. *Msx1b*, *Fgf10*, *Fgf8*, and *Prrx1* were positive in both the bump and the regular blastema, while they were negative in intact skin. *Hoxa13* could not be detected in the bump but could be detected in the regular blastema. *Bmp* genes were also investigated by RT‐PCR. *Bmp2*, *4*, and *7* were positive in both the bump and the regular blastema. *Type II collagen* and *Sox9* were unexpectedly detectable in the bump, but the regular blastema showed much higher expression level. *Ef1α* was an internal control.

To investigate the gene expression pattern of these bumps, reverse transcription polymerase chain reaction (RT‐PCR) analysis was performed (Fig. 3). *Msx1*, which is exclusively expressed in undifferentiated blastemal mesenchyme, was detected, as were *Fgf10* and *Fgf8*, which are regulated by a positive feedback loop during limb development and presumably during regeneration (Yokoyama et al. 2000; Martin 2001; Suzuki et al. 2005). *Prrx1* expression was consistent with the in situ hybridization results shown in Fig. 2D. Although *Hoxa11* could be detected, *Hoxa13* was undetectable in the induced bump by RT‐PCR. *Bmp* genes (*Bmp2*, *Bmp4*, and *Bmp7*) were detectable in the bump. *Sox9* and *type II collagen* expressions, which can be observed in cartilage cells, were also investigated. Regenerating blastemas in amputated limbs expressed both genes at high level. Unexpectedly, both genes were detected in the bump. This would be because of tissue contexts. *Sox9* is expressed in keratinocytes (Shi et al. 2013), which is consistent with *Sox9* detection in the skin sample. Furthermore, glial cells are reported to express *type II collagen* (D'Antonio et al. 2006). The bump contained many nerve bundles and associated glial cells. Therefore, it is likely that RT‐PCR detected non‐cartilaginous *Sox9* and *type II collagen* expressions. In general, gene and protein expression patterns were similar to those reported in the axolotl ALM, although more nerve fibers were necessary for induction.

### Accessory blastemas in *Xenopus* do not have cartilage differentiation capacity

**Figure 4:**
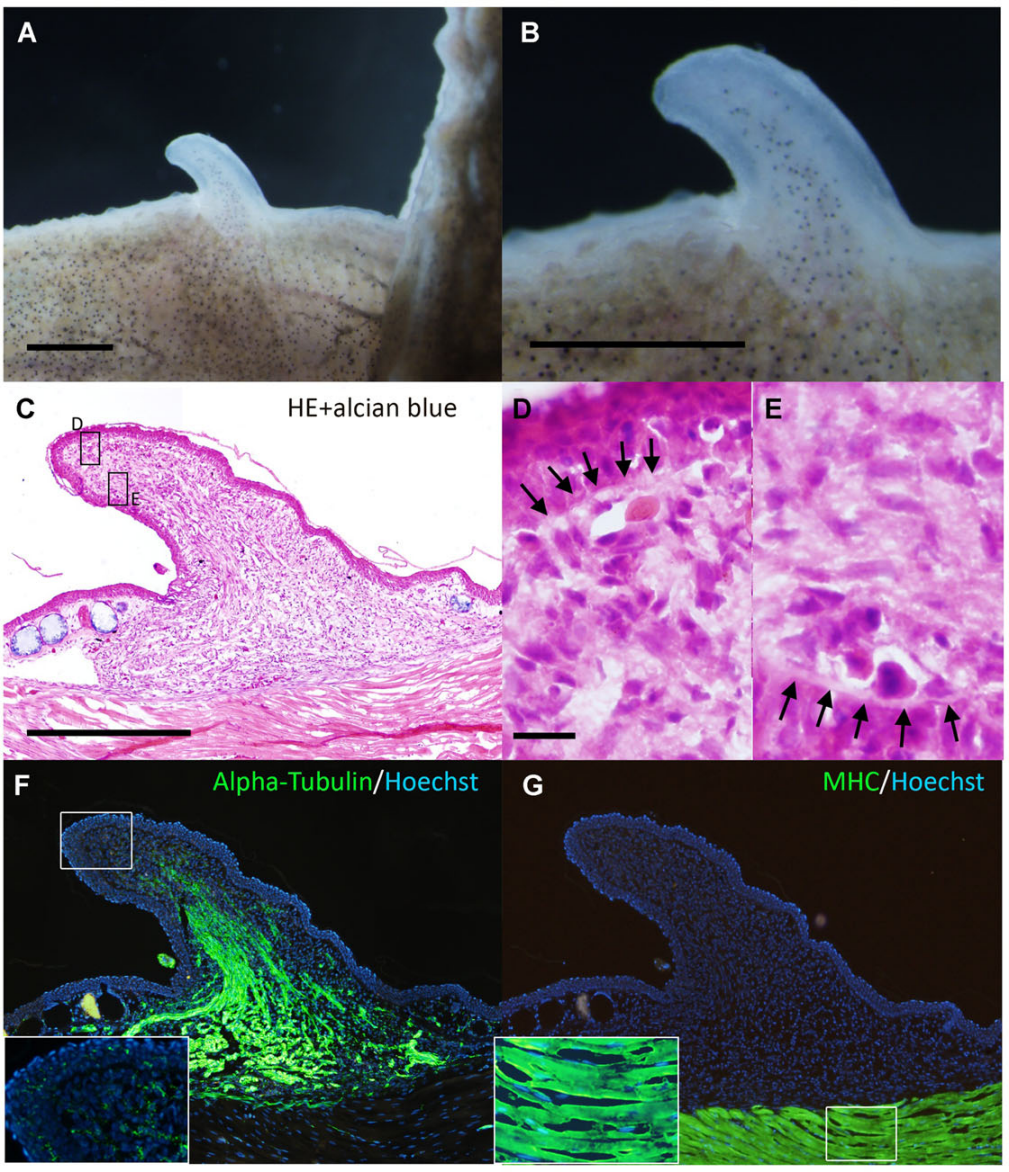
The induced bump did not contain cartilaginous cells. (A, B) Dorsal views of the day 20 bump. The induced bump stopped growing at day 20. (C) Histology of the bump. There are no Alcian‐blue‐positive cartilage cells in the mesenchymal region. (D, E) Higher magnification of the boxed regions in (C). Arrows in (D) and (E) indicate developing basal laminae. (F) Axons were visualized by immunofluorescence using anti‐acetylated alpha‐tubulin. In the distal region, highly innervated axon fibers in the epithelium were not observed in the day 20 bump (inset). (G) Myosin heavy chain (MHC) expression. There were no MHC‐positive myogenic cells in the bump. Scale bars in (A) and (B) are 1 mm. The scale bar in (C) is 500 μm. (C), (F), and (G) are the same magnification. The scale bar in (D) is 20 μm. (D) and (E) are the same magnification.

In the axolotl ALM, the induced ectopic blastema shows cartilage differentiation capacity even in the absence of a skin graft (Endo et al. 2004; Satoh et al. 2010a). However, all *Xenopus* bumps stopped growing before cartilaginous spike formation and started to regress (Table 1). The induced blastemas were fixed at near maximum size (Fig. 4A, B) and sectioned for histological analysis, which revealed no Alcian‐blue‐positive cartilage cells inside the blastema (Fig. 4C–E). Consistent with the limited growth of *Xenopus* bumps, a basement membrane developed at the distal region of the mesenchyme–epithelium border (Fig. 4D, E). Axon fibers were still present in abundance in the induced structure at growth termination (Fig. 4F), but myogenic cells were undetectable (Fig. 4G). These observations suggest that the induced bump does not possess cartilage differentiation capacity and cannot recruit muscle cells.

### Accessory blastemas in *Xenopus* grow spikes only when bone is damaged

**Figure 5:**
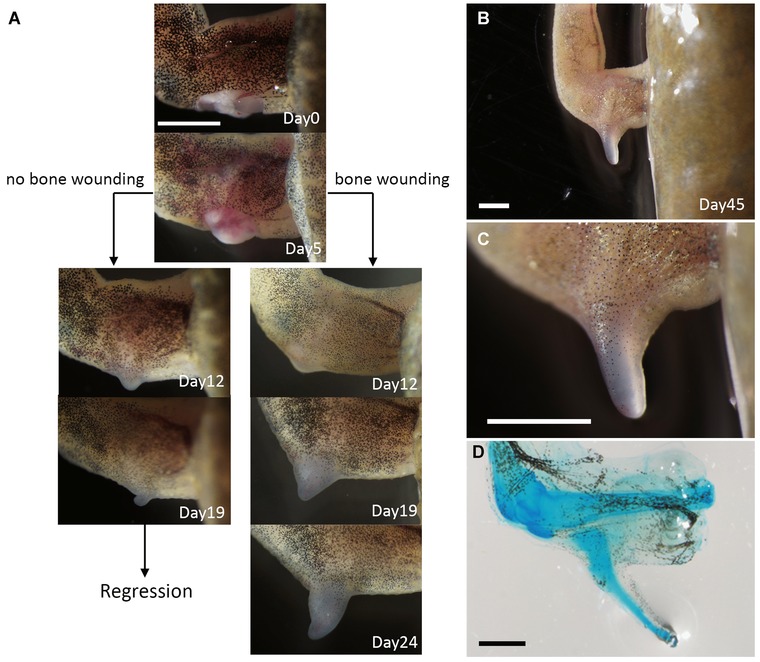
Cartilaginous spike induction by deep wounding. (A) Time course of the induced bump and spike induction. Skin wounding plus nerve deviation result in bump formation. In this case, a small bump can be seen within 12 days, but it starts regressing by day 19. On the other hand, when a deep wound is created enough to damage a stylopod bone, a cartilaginous spike can be observed. In this case, a relatively smaller bump can be observed at day 12. The induced bump kept growing up to spike formation. (B, C) Induced cartilaginous spike at day 45. (D) Whole‐mount Alcian blue staining. Scale bars in (A), (B), (C), and (D) are 2 mm.

**Figure 6:**
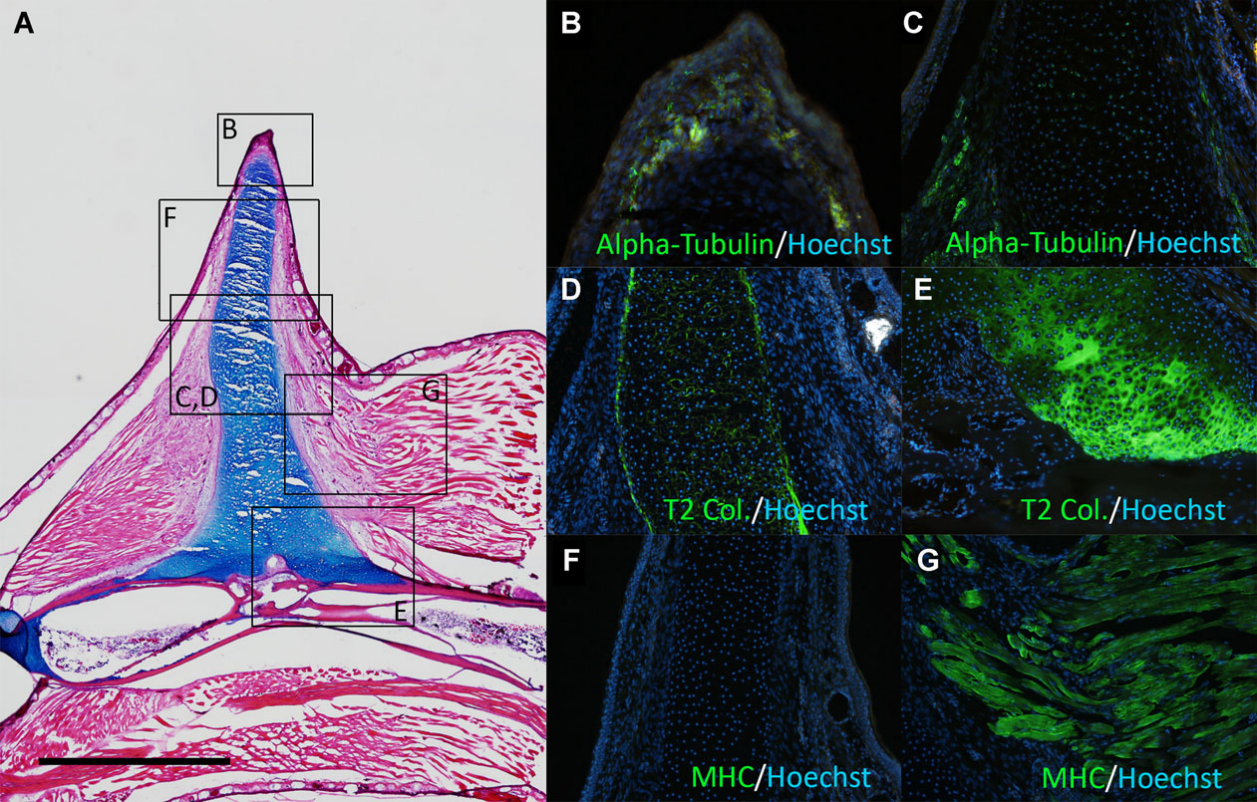
Section of the cartilaginous spike induction by deep wounding. (A) Histological observation. Alcian‐blue‐positive cartilage extends from the stylopod. (B, C) Acetylated alpha‐tubulin expression. (D, E) Type II collagen (T2 Col.) expression. (F, G) MHC expression. (B)−(G) are the boxed region of (A). The scale bar in (A) is 1 mm.

In the previous axolotl ALM study, two different regeneration systems were proposed, one AEC dependent and the other AEC independent (Satoh et al. 2010b). The regular ALM surgery activates the AEC‐dependent system, while the AEC‐independent system can be activated by damage to deeper tissues such as bone (Satoh et al. 2010b). Thus, a similar surgery was performed on *Xenopus*. The stylopod bone (humerus) was cracked and the surrounding muscle tissue was removed during the ALM surgery as described previously for axolotls (Satoh et al. 2010b). When all major nerve bundles had been deviated to the deeply wounded site, spike formation could be observed (Table 1, Fig. 5), although the induction was slower than the induction of the bumps following regular ALM surgery (Fig. 5A). While bumps appearing in the absence of deep tissue damage grew to a maximum and then regressed without cartilage expression, those induced by combined ALM surgery and bone damage formed bumps that continued to grow (Fig. 5A). Approximately 45 days later, a cartilaginous spike could be seen (Fig. 5B–D). Alcian blue staining revealed no joint formation, consistent with previous reports (Fig. 5D; Endo et al. 2000; Satoh et al. 2006). Although most of the induced spike cartilage projected from the stump bone, some were not continuous with the stump bone but originated from the deeper regions of the limb (Fig. 5D). To delineate the structure of the induced spike, samples were sectioned and examined by immunofluorescence (Fig. 6). Histological analysis reveals that the spike cartilage extended from the humerus (Fig. 6A). The calcified bone was Alcian blue negative and the cartilaginous cells extended toward the induced bump (Fig. 6A). Axon fibers were detected in both proximal and distal regions (Fig. 6B, C) but the distal epithelium no longer contained abundant axon fibers (Fig. 6B). Also, type II collagen was clearly detected in the spike cartilage (Fig. 6D, E). As expected, no muscle cells were detected in the spike region but could be seen in the proximal (intact) region (Fig. 6F, G). This expression pattern is comparable with that observed in regular limb‐amputation‐induced spikes (Satoh et al. 2005).

## Discussion

### Conserved nerve functions in limb regeneration

*Xenopus laevis* possesses only partial limb regeneration capacity but, like the axolotl (a species with full limb regenerative capacity), adult *Xenopus* can grow accessory blastema‐like formation (bump) when peripheral nerves are rerouted to a skin wound. Moreover, these *Xenopus* bumps were structurally comparable with those induced by the axolotl ALM procedure and showed a similar gene expression pattern (Figs. 1–3), indicating that many of the mechanisms responsible for early blastema development are conserved between these species. However, whether a blastema on an amputated *Xenopus* limb is a “true” blastema is debatable since it grows into a spike rather than a limb. Nonetheless, the early parallels in blastema development suggest that similar or the same nerve‐derived factors coordinate the early phases of regeneration following limb amputation, including induction of AEC and blastema cells, in both urodele and anuran amphibians. During the early stages of blastema development, the only notable difference was the requirement for multiple nerve bundles in *X. laevis*. In axolotls, deviation of only one of three major brachial nerve bundles was usually sufficient to induce blastema formation, while at least two nerve bundles were necessary to induce an ALM response in *Xenopus* (Fig. 1). This difference in tissue requirements may be due to lower concentrations or reduced sensitivities to nerve factors that induce blastema formation. In fact, grafting of ectopic nerve tissue promoted limb regeneration in anuran amphibians following amputation (Singer 1954). These hyperinnervated structures appeared more “limb‐like” than spikes, although the complete limb was not regenerated. If lower levels of specific neural factors were the only cause of reduced regenerative capacity of anuran amphibians, it should be possible to augment the supply sufficiently to induce complete regeneration, as occurs in newts and axolotls. To date, no such demonstration has been observed, which suggests that additional factors, possibly supplied by urodele nerves but not anuran nerves or another tissue type, are necessary. Nonetheless, several candidate nerve‐derived factors have been isolated (Nye et al. 2003) that may be used in human regenerative medicine.

### Implications of directed cartilaginous extension from the stump bone

In the present study, we demonstrated that nerve deviation to the site of a skin wound did not give rise to cartilaginous cells in *Xenopus*. Rather, the bump consisted mainly of fibroblastic cells and axons (Fig. 4C–F). In contrast, axolotl blastemas induced using the same ALM procedure eventually have cartilage cells (Endo et al. 2004). Two potential possibilities that cause the difference in cartilage differentiation capability can be considered. First, the absence of some kinds of nerve factors leads to failure of cellular dedifferentiation. Indeed, larger numbers of axon bundles were necessary for bump induction in *Xenopus* than in axolotl as mentioned above. This suggests a shortage of nerve factors in each nerve bundle in *Xenopus*. In the present study, three major nerve bundles, which are the maximum numbers of reroutable nerves in limb, were used for bump induction. Therefore, to make up for the shortage of nerve factors, it is necessary to identify nerve factors in *Xenopus*. Second, an intrinsic restriction inhibits cellular dedifferentiation even though nerve factors are the same and of sufficient amount. In this case, comparative analysis between axolotl/newt cells and *Xenopus* cells would be helpful. Anyway, acquiring cells having cartilage‐differentiating ability is the key to understanding the differences in regeneration ability in *Xenopus* and axolotl.

In *Xenopus*, cartilage‐containing bumps were obtained only if the humerus was damaged to activate bone healing processes (Fig. 6A). In this case, the cartilage extended from the stump bone to the bump (Fig. 6A). The bone was not segmented or branched, and the morphology was similar to a spike raised by an amputated limb (Satoh et al. 2005). If the induced structure is a directed cartilage extension, two possibilities can be considered: (1) a damaged bone releases cartilaginous progenitor cells for forming a spike; and (2) bone damage releases growth and differentiation factors for cartilage formation that are normally present in axolotls in the absence of bone damage. Further studies are necessary to investigate this.

Cartilaginous callus formation is observed in response to bone damage across species, including humans. In the case of higher vertebrates, calcification follows immediately after cartilage formation (Ide 2012; Yu et al. 2012). Continuous input of Bmp2 or Bmp7 directs cartilaginous extension, resulting in digit/arm regeneration (Ide 2012; Yu et al. 2012). Bone‐damage‐induced spike formation in *Xenopus* has many similarities to mouse limb/digit regeneration models requiring exogenous Bmp application (Ide 2012; Yu et al. 2012). However, this process is autonomously initiated in *Xenopus*. Thus, *Xenopus* possess a regenerative capacity which is intermediate between that of higher vertebrates and urodele amphibians.

## Materials and methods

### Animals and surgical procedures

Adult *X. laevis* was obtained from domestic animal venders and maintained at 20–22°C in dechlorinated water. Animals were anesthetized using 0.1% ethyl 3‐aminobenzoate methanesulfonate salt (Sigma, St. Louis, MO, MS222), pH 7.0, for surgical procedures.

Accessory limb model surgery was performed as previously described (Endo et al. 2004; Makanae and Satoh 2012). To induce ectopic blastemas, a square section of skin (1.0−1.5 × 2−3 mm) was carefully removed from the posterior side of the upper arm without damaging the underlying muscle. A ventral incision was made from the shoulder to the elbow, and the brachial nerve was dissected free and severed at the elbow level. The nerve was rerouted beneath the skin to bring the cut end to the center of the skin wound. Similarly, nerves were rerouted to the wound from the dorsal side. A square section of skin (1.0−1.5 × 1.0−1.5 mm) was removed from the anterior lower arm and grafted to the site of the skin wound.

### Histology

Samples were fixed in 4% paraformaldehyde for a day. Cryosections were prepared with a thickness of 10 μm. Air‐dried tissue sections were immersed in tap water to remove the optimal cutting temperature compound (Sakura), stained with Alcian blue solution (Wako, Osaka, Japan) for 3 min, washed with water, stained with hematoxylin (Wako) for 5 min, washed with tap water for several minutes, stained with eosin (Wako) solution for 5 min, and finally washed in 70% ethanol. Sections were then dehydrated with ethanol and mounted using Softmount (Wako).

To visualize the cartilage of the regenerated skeletal elements in whole‐mount preparations, samples were fixed overnight in 10% neutral buffered formalin solution (Wako), incubated in 70% ethanol plus 1% HCl for 3–4 h, and stained with 0.1% Alcian blue in 70% ethanol plus 1% HCl for 2–3 days. Samples were then washed with 4% KOH without agitation for 2 h at room temperature (RT), incubated in a solution of 2% KOH/50% glycerol for 1–3 days, and cleared in 100% glycerol prior to photography.

### Immunohistochemistry

Immunohistochemistry of tissue sections was performed as previously described (Satoh et al. 2007) using anti‐acetylated alpha‐tubulin (Sigma, 1/200), anti‐fibronectin (Sigma, 1/100), anti‐type II collagen (DSHB, 1/100), anti‐MHC (DSHB, Iowa City, IA, 1/50), anti‐rabbit IgG‐Alexa 488 (Invitrogen, Karlsruhe, Germany), and anti‐mouse IgG‐Alexa 488 (Invitrogen). Nuclei were counterstained with Hoechst 33258 (Dojindo, Kumamoto, Japan) and images were captured using an Olympus BX51 microscope.

### Reverse transcription polymerase chain reaction (RT‐PCR)

Total RNA was isolated from regenerating blastemas and intact skin from forelimbs and hindlimbs using TriPure reagent (Roche, Mannhein, Germany). Total RNA was used as a template for first‐strand cDNA synthesis using oligo (dT) primers. Primescript reverse transcriptase (Takara, Otsu, Japan) was used for the extension according to the manufacturer's instructions. Each PCR cycle was performed as follows: 96°C for 15 sec, 58°C for 30 sec, 72°C for 60 sec, and a final extension for 5 min at 72°C. We performed 28 cycles for *Msx1b*, *Bmp2*, *Bmp4*, *Bmp7*, *Sox9*, and *type II collagen* and 30 cycles for *Fgf10*, *Fgf8*, *Prrx1*, *Hoxa11*, *Hoxa13*, and *Ef1α*. The following primers were used: *Msx1b* forward, ATGGCCCCGGCTCTGCTTATGGC; *Msx1b* reverse, TTAGGACAGATGGTACATGCTG; *Fgf10* forward, ATGTTGGTGCCTTATTCTCTGC; *Fgf10* reverse, TTATACAACAATTGGAAGAAAATG; *Fgf8* forward, ATGAACTACATCACCTCCATCC; *Fgf8* reverse, CTACCGAGAACTTGAATATCG; *Prrx1* forward, ATGACCTCCAGCTATAACCACC; *Prrx1* reverse, TCAATTGACTGTTGGCACTTGG; *Hoxa13* forward, ATGACAGCTTCAGTGCTCCTCC; *Hoxa13* reverse, TTAACTCGTACTTTTCAGTTTA; *Hoxa11* forward, TACCAGATTAGAGAACTGG; *Hoxa11* reverse, CACTGTGCGCAATTAACTTG; *Bmp2* forward, CCATGAAGAATCCATGGAAGA; *Bmp2* reverse, GGCAACAATCCAGTCATTCC; *Bmp4* forward, AGCCCAGTAAGGATGTGGTG; *Bmp4* reverse, TTTTTACGGGGTCTCTGCTG; *Bmp7* forward, GCCCCTATGTTTATGCTGGA; *Bmp7* reverse, TCTTGGCTCTTTGGTGCTTT; *Sox9* forward, AACAAGCCCCATGTCAAGAG; *Sox9* reverse, ATGACTTGGGCTCAGTTGCT; *type II collagen* forward, GACGTGAAGGTAACCCTGGA; *type II collagen* reverse, CAGTCTCCATGTCGCAGAAA; *Ef1α* forward, CTGGAACCTCTCAGGCAGAC; *Ef1α* reverse, TCTGCGAATGTCCTTGACTG.

### In situ hybridization

To synthesize antisense RNA probes, templates were synthesized using PCR with Ex Taq DNA polymerase (TaKaRa) and transcribed with T7 RNA polymerase (for *Prrx1*, TaKaRa). Specimens were fixed overnight at RT in 4% paraformaldehyde/phosphate‐buffered saline and then decalcified in 10% ethylenediamine tetra‐acetic acid at RT. Samples were treated with 5 μg/mL Proteinase K, refixed in 4% paraformaldehyde/phosphate‐buffered saline, and hybridized overnight at 63°C in a solution containing RNA probes. After hybridization, the sections were washed in 50% formamide, twice in 5× saline−sodium citrate (SSC) for 30 min at 63°C, again in 50% formamide, three times in 1.5 × SSC for 30 min at 63°C, and finally in TBST (10 mmol/L Tris–HCl, 150 mmol/L NaCl, 0.1% Tween 20) at RT before blocking in 0.5% blocking reagent (Roche) for 30 min. The samples were then incubated with alkaline phosphatase (AP) conjugated anti‐digoxigenin‐AP antibody (Roche, 1/1000) for 2 h at RT and then washed three times with TBST (10 min/wash). Immunolabeling was visualized using nitroblue tetrazolium chloride (Wako) and 5‐bromo‐4‐chloro‐3′‐ indolylphosphatase *p*‐toluidine salt (Wako) as a substrate for AP in a buffer containing 100 mmol/L NaCl, 100 mmol/L Tris (pH 9.5), 20 mmol/L MgCl_2_, and 0.001% Tween 20.
